# Transcriptional stress in aging: integrating experimental data and modeling to quantify DNA damage accumulation

**DOI:** 10.3389/fmolb.2025.1659589

**Published:** 2025-09-08

**Authors:** Jacinta van de Grint, Marko Raseta, Renata Brandt, Yvette van Loon, Joris Demmers, Shannon Dealy, Jiang Chang, Jan Hoeijmakers, Joris Pothof

**Affiliations:** ^1^ Department of Molecular Genetics, Erasmus MC Cancer Institute, Erasmus Medical Center, Rotterdam, Netherlands; ^2^ Princess Maxima Center for Pediatric Oncology, Oncode Institute, Heidelberglaan, Netherlands; ^3^ Faculty of Medicine, Cluster of Excellence for Aging Research, Institute for Genome Stability in Ageing and Disease, University of Cologne, Cologne, Germany

**Keywords:** aging, transcriptional stress, DNA damage, mathematical modeling, nucleotide excision repair

## Abstract

Accumulating DNA damage plays a crucial role in aging, particularly in post-mitotic tissues by disrupting transcription and causing transcriptional stress—a state marked by reduced transcriptional productivity. Transcriptional stress disproportionately affects long genes, due to the random distribution of DNA lesions across the genome. An estimate for the total number of transcription-blocking lesions (TBLs) required to induce transcriptional stress and contribute to aging is lacking. Here, we estimated the number of TBLs accumulating with age, by integrating experimental data with a mathematical model based solely on fundamental biological principles. Using 5-ethynyluridine (EU) incorporation, we assessed transcriptional activity in dermal fibroblasts and liver tissue from *Ercc1* ^−/−^, *Ercc1* ^*d*/-^, and *Xpg* ^−/−^ mice—models with DNA repair deficiencies that exhibit a wide range of premature aging features between 5 and 26 weeks of age. We then compared the experimental data to our model, which captured the overall trend of transcriptional decline, supporting a correlation between accumulating DNA damage and reduced transcription during aging. Wildtype mice were found to accumulate approximately 62 TBLs per day, whereas DNA repair-deficient mice exhibited a markedly higher burden, accumulating 1,600–5,000 TBLs daily. These insights offer a quantitative understanding of transcriptional stress, which is crucial for elucidating the aging process.

## 1 Introduction

Aging is generally associated with a progressive decline in physical and cognitive abilities, and a key role in this process is played by DNA damage (Schumacher et al., 2021; Hoeijmakers, 2009). DNA lesions are generally very difficult to detect and reliably quantify because of their heterogeneous nature and low abundance. However, it is estimated that mammalian cells may encounter on average approximately 100,000 DNA lesions daily from endogenous and exogenous sources, with roughly 10^4^ being AP sites—abasic sites where a DNA base has been lost due to spontaneous hydrolysis. (Schumacher et al., 2021). Ultraviolet (UV) radiation from sunlight alone can already lead to the formation of up to 100,000 lesions per day in keratinocytes (Hoeijmakers, 2009), underlining the substantial burden of DNA damage on the genome. While cells possess a sophisticated network of DNA repair pathways to counteract various types of damage, these mechanisms are not 100% efficient. Consequently, DNA damage accumulates in tissues over time, contributing to the aging process (Schumacher et al., 2021; Hoeijmakers, 2009; Yousefzadeh et al., 2021; Lombard et al., 2005).

As DNA damage accumulates in the genome with age (Schumacher et al., 2021; Yousefzadeh et al., 2021; Lombard et al., 2005), it physically blocks elongating RNA, thereby interfering with gene expression (Gyenis et al., 2023). Moreover, a lesion-stalled RNA polymerase in the liver of a 2-year old wildtype mouse is estimated to arrest on average three additional RNA polymerases, forming a queue (Gyenis et al., 2023). In cells with a high number of transcription-blocking lesions (TBLs), this results in reduced transcriptional productivity, a state known as transcriptional stress. Through this mechanism, DNA damage can impact nearly every hallmark of the aging phenotype, such as genomic instability, loss of proteostasis, deregulated nutrient sensing and inflammation (López-Otín et al., 2023), suggesting it could serve as a unifying driver of the aging process (Schumacher et al., 2021; Gyenis et al., 2023).

Since DNA damage occurs roughly uniformly across the genome, long genes statistically face damage more frequently than short genes. This skews transcriptional output toward shorter genes, impacting gene expression profiles (Gyenis et al., 2023). Such shifts in transcriptional activity disrupt the regulation of numerous pathways associated with various aging hallmarks (Gyenis et al., 2023). Our lab identified transcriptional stress in multiple species and tissues: in at least 14 different tissues in mice, 11 in rats, and 6 in humans, ranging from heart to retina, and provided direct evidence that it is caused by DNA damage (Gyenis et al., 2023).

Other studies have also reported gene-length dependent transcriptional stress (Soheili-Nezhad et al., 2024; Soheili-Nezhad et al., 2021; Stoeger et al., 2022; Ibañez-Solé et al., 2023; Vermeij et al., 2016). For instance, Stoeger et al. further identified increased transcriptional stress with age in various transcriptomic datasets from mice and humans, and found that the phenomenon is responsive to anti-aging interventions (Stoeger et al., 2022). Additionally, Ibañez-Solé et al. observed transcriptional stress in single-cell RNA sequencing datasets across multiple tissues and cell types during mouse and human aging (Ibañez-Solé et al., 2023). Enrichment analyses indicate that long genes are indeed associated with longevity and aging-related pathways (Gyenis et al., 2023; Stoeger et al., 2022). Finally, Soheili-Nezhad et al. found that long genes are less expressed in brains of Alzheimer’s patients, potentially contributing to the disease’s pathogenesis (Soheili-Nezhad et al., 2021). This transcriptional stress was more pronounced in brain regions commonly affected in Alzheimer’s disease, and less in regions more resilient to Alzheimer’s disease (Soheili-Nezhad et al., 2021). These findings collectively indicate that transcriptional stress likely plays an important role in the aging process, though causality has not yet been established.

The connection between transcriptional stress and aging is well-illustrated by progeroid mouse models, such as *Ercc1* ^*d*/-^ and *Xpg* ^−/−^ mice (Gyenis et al., 2023; Vermeij et al., 2016). These mouse models have genetic deficiencies in either *Ercc1* or *Xpg*, factors that are crucial for repairing DNA lesions that impede transcription via transcription-coupled nucleotide excision repair (TC-NER) (Lans et al., 2012). They exhibit progressively reduced transcription levels in tissues like the liver and kidney (Gyenis et al., 2023), alongside numerous characteristics associated with premature aging, such as declining liver, kidney and cardiovascular function, weight loss, sarcopenia, and features of neurodegeneration including tremors, hearing and vision loss, imbalance and overall frailty (Gregg et al., 2011; Niedernhofer et al., 2006; Barnhoorn et al., 2014).

As estimated in our previous analysis, approximately 40% of elongating RNA polymerases appear stalled in liver of a 2-year old wildtype mouse (Gyenis et al., 2023). This degree of transcriptional stress is most likely caused by various types of endogenous DNA lesions which are capable of blocking transcription, including cyclopurines, DNA adducts of aldehydes, interstrand crosslinks, DNA-protein crosslinks (van Sluis et al., 2024) and advanced glycation end products (AGEs) (Tamae et al., 2011; Tiwari et al., 2019). However, the quantity of DNA lesions responsible for inducing transcriptional stress and contributing to aging has only been approximated for some lesion types.

In this study, our objective was to estimate the number of TBLs that form during aging by integrating experimental data with a mathematical model. Initially, we quantified the level of DNA damage that leads to a certain reduction in transcription and assessed the accuracy of this method. We investigated global transcription levels using EU incorporation in human fibroblasts following UV light treatment, which induces transcription-blocking DNA damage, and compared the results to the predictions of our mathematical model. In addition to cultured cells, we analyzed global transcription levels in mice. We used liver tissue slices obtained from wildtype, *Ercc1* ^−/−^, *Ercc1* ^*d*/-^, and *Xpg* ^−/−^ mice across various ages to assess the accumulation of DNA damage associated with aging *in vivo* and compared this data with our mathematical model. We found notable similarities between the experimental data and the model, as well as some remarkable differences. Our study provides a quantitative framework for estimating TBLs during aging, offering insights into how transcriptional stress accumulates over time.

## 2 Results

### 2.1 Model assumptions

**FIGURE 1:**
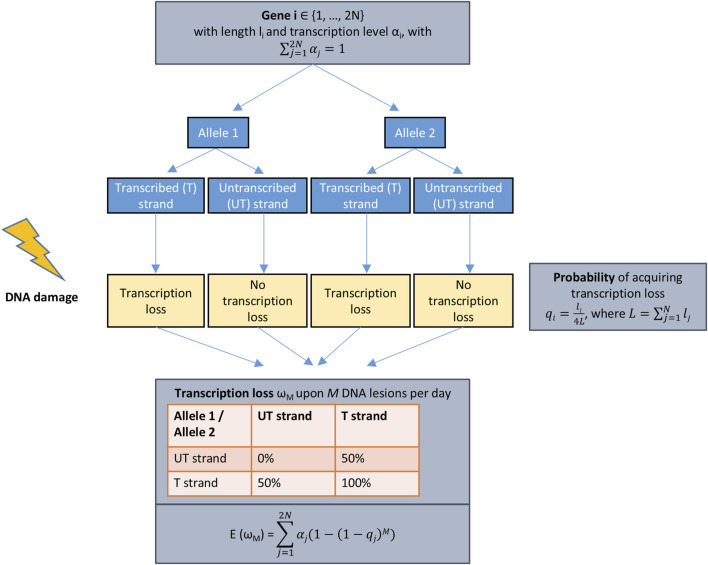
Schematic of the mathematical model. Flow diagram of the mathematical model of transcription loss. A gene i with length $l_{i}$ and transcription level $\alpha_{i}$ has a certain probability $q_{i}$ of acquiring transcription loss, which is proportional to its length. The expected value of transcription loss $E\left(\omega_{M}\right)$ was derived in (Raseta et al., 2024) using the model’s assumptions: 1) Persistent DNA damage occurs with a certain rate in both strands. 2) Each gene has two alleles on different homologous chromosomes, and transcription is evenly distributed between them. 3) DNA damage occurs both in the transcribed (T) strand and the untranscribed (UT) strand. If DNA damage occurs in the UT strand, it does not lead to transcription loss, whereas in the T strand, it does.

We developed a mathematical model to better understand the characteristics of DNA damage accumulation and transcriptional stress over time, allowing us to estimate the approximate number of TBLs accumulating with aging (Figure 1). The mathematical model, elaborated in detail in (Raseta et al., 2024), is based on four biological assumptions. First, assuming a diploid state, transcription is evenly distributed between the two alleles of a gene present on the pair of homologous chromosomes. Second, DNA damage is equally likely to occur in either of the two DNA strands, so when DNA damage occurs, there is a 50% chance that transcription will be maintained. Third, our model exclusively considers unrepaired (i.e., persistent) DNA damage, ignoring DNA lesions that are repaired. Although repair is not considered in the model, the framework remains applicable to organisms with functional repair mechanisms, as DNA repair acts to reduce, but not entirely prevent, the accumulation of TBLs. In this case, DNA damage is simply modeled to accumulate at a slower rate. Finally, persistent DNA damage is uniformly distributed across the genome and accumulates at a constant, defined input rate, as supported by quantifications of different DNA lesion types in different mouse tissues (Osterod et al., 2001) and human PBMCs (Vlachogiannis et al., 2023). Although specific hotspots have been identified (Amente et al., 2021; Wu et al., 2021), possibly due to factors like chromatin structure, this assumption of uniform distribution is also the basis of another mathematical model on aging cells (Song and Acar, 2019). This approach allows us to account for the cumulative accumulation of unrepaired DNA damage in each cell over an organism’s lifespan.

As input, the model requires a list of genes expressed in the specific organ and cell type of the target species. For each gene i *∈ {1, … , 2N}*, it also requires its length $l_{i}$ to calculate the probability of acquiring TBLs $q_{i}$ . Since we assume DNA damage is uniformly distributed across the genome, the probability $q_{i}$ is proportional to the gene’s length $l_{i}$ and the total genome length L. Since there are two alleles of each gene, with each allele consisting of two DNA strands, L is multiplied by 4. Therefore, the probability $q_{i}$ is given by (Raseta et al., 2024):

$$q_{i}=\frac{l_{i}}{4L}$$

Additionally, the model requires the weighted expression level $\alpha_{i}$ for each expressed gene, measured by nascent RNA sequencing, with $\sum_{j=1}^{2N}\alpha_{j}=1$ (Figure 1).

### 2.2 Transcription in human fibroblasts after UV damage

It is essential to understand the variability of *in vitro* data before applying our model to *in vivo* data, as the intrinsic error will be amplified in *in vivo* data. Therefore, we first calibrated the model, described in more detail in (Raseta et al., 2024), for human dermal fibroblasts using expression data. To this end, we sequenced nascent RNA from untreated human wildtype C3RO dermal fibroblasts (N = 4 samples) and acquired a list of expressed genes along with their expression levels (Supplementary Figures 1A, B; Supplementary Table S1). Here, the variable M , representing the number of TBLs, is treated as the independent input variable, meaning it is set manually and determines the output of the model: the expected value of transcription loss $E\left(\omega_{M}\right)$ :

$$E\left(\omega M\;\right)=\sum_{j=1}^{2N}\alpha_{j}\left(1-{\left(1-q_{j}\right)}^{M}\right)$$

as derived in Proposition 2 of (Raseta et al., 2024). We used a human XPA-deficient dermal fibroblast cell line named XP25RO to ensure that DNA damage induced by UV remains unrepaired, as both global genome-nucleotide excision repair (GG-NER) and TC-NER are dependent on XPA (van den Heuvel et al., 2023; Manandhar et al., 2017). Importantly, despite the inability to repair DNA damage in XPA-deficient cells, global transcription in untreated condition remains unaffected (Manandhar et al., 2017). This approach enables accurate quantification of transcription loss attributed to a defined DNA damage dose.

Cells were synchronized in G0/G1 by growing to full confluency and then culturing for an additional 2 weeks before UV treatment. This extended culturing period ensured that only a minor, negligible fraction of cells continued proliferating, which helped avoid cell death during S-phase caused by the induced damage and prevented DNA repair pathways active solely in S-phase from repairing the damage. The percentage of replicating cells, quantified using EdU (5-ethynyl-2-deoxyuridine) incorporation, never exceeded 7% in any sample and was below 2% in most samples (Supplementary Figure S2A).

We induced transcriptional stress in cells using UV-C irradiation, because it induces TBLs in a quick and constant manner, and the amount of TBLs induced per 1 J/m^2^ UV-C exposure is known (Kalinowski et al., 1992; Andrade-Lima et al., 2015; Bohr et al., 1985). After UV treatment, cells were incubated for an additional 24 h before EU was added for 1 h. This 24-h time point was chosen to avoid capturing the acute transcriptional shutdown that occurs immediately after UV exposure, and to instead assess persistent transcriptional defects. Our analysis of a previously published nascent RNA sequencing dataset (Andrade-Lima et al., 2015) showed that, by 24 h post-UV, transcription levels and the intragenic distribution of RNA sequencing reads in wildtype cells had returned to baseline (Supplementary Figures S3A, B). At this time point, transcriptional tilt toward the 5′end—caused by polymerase stalling at TBLs—had also resolved in GG-NER-deficient (XPC^−/−^) cells (Supplementary Figures 3C, D), but persisted in TC-NER-deficient (CSB^−/-^) cells (Supplementary Figures 3E, F), where stalled polymerases obstruct lesion repair despite partial recovery of average transcript levels. In this study, we used XPA-deficient cells, which lack both TC-NER and GG-NER activity. Similar to CSB-deficient cells, XPA-deficient cells likely retain persistent transcriptional asymmetry, despite resolution of the acute, global transcriptional repression by 24 h.

**FIGURE 2:**
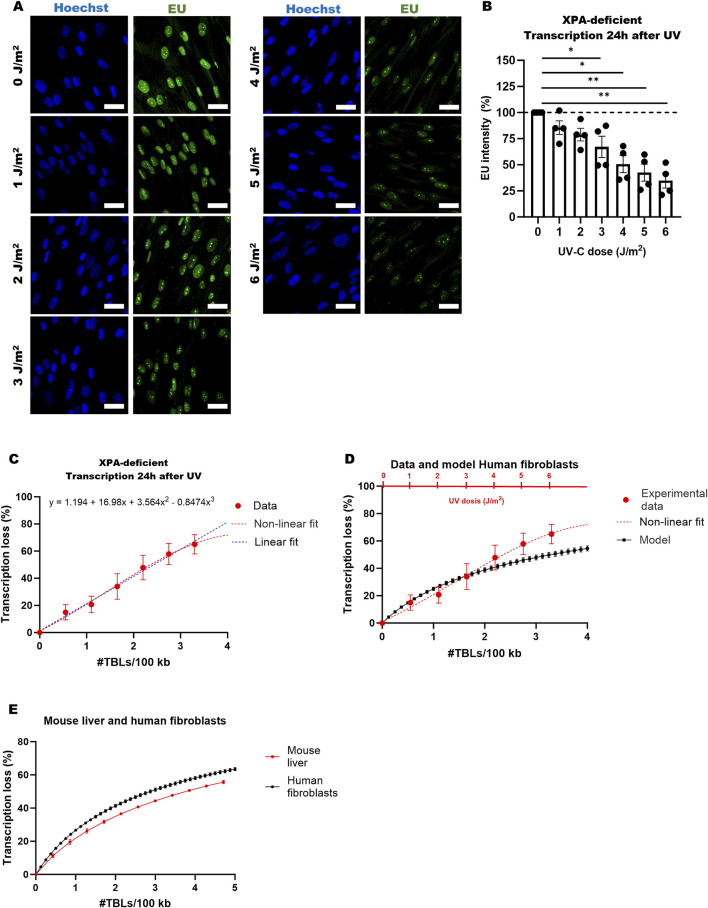
Transcription in human fibroblasts after UV-induced DNA damage. **(A)** Representative confocal images of XP25RO (XPA-deficient) cells treated with different UV doses (0–6 J/m^2^), stained with transcription marker EU (green) and DNA marker Hoechst (blue) 24 h post-treatment. Scale bar = 30 μm. **(B)** Quantification of EU intensity in XP25RO cells 24 h after UV exposure (0–6 J/m^2^), based on four independent experiments. Error bars represent the SEM. Unpaired two-tailed Student’s t-test: *p ≤ 0.05; **p ≤ 0.01; ***p ≤ 0.001; ****p ≤ 0.0001. **(C)** Non-linear (third-order polynomial) and linear regression analysis of EU intensity data from four independent experiments in XP25RO cells. EU intensity was converted to transcription loss (100% – EU intensity), and UV dose was converted to amount of TBLs per 100 kb. Error bars represent the SEM. **(D)** Mathematical model on transcriptional stress in human cells, depicting transcription loss $E\left(\omega_{M}\right)$ versus DNA damage per 100 kb, together with experimental data and non-linear fit from **(C)** in red. Transcription loss was modelled for different doses of DNA damage in between 0 and 4 DNA lesions per 100 kb to generate the black curve. Black error bars represent the 95% confidence interval of the model calibrated on nascent RNA sequencing data from four independent cell populations. Red error bars depict the SEM of four independent experiments. **(E)** Transcription loss $E\left(\omega_{M}\right)$ as a function of the number of TBLs M per 100 kb, as predicted by the mathematical model for both mouse liver and human fibroblasts. In both input data sets, genes below 5 kb were filtered out. Error bars indicate the standard deviation.

We observed a UV dose-dependent decrease in EU-intensity levels by approximately 15% after exposure to 1 J/m^2^ and by about 65% after 6 J/m^2^ (Figures 2A,B). Our focus was exclusively on quantifying the EU signal within the nucleoplasm, i.e., the nucleus without nucleoli, to avoid bias from high, damage-resistant rRNA transcription. In the context of aging, low doses (below 2 or 3 TBLs per 100 kb) are of physiological relevance, as such levels are typically encountered under natural conditions, such as chronic low-level UV exposure combined with endogenous DNA damage (Lee et al., 2020). Given that 1 J/m^2^ translates to approximately 0.55 lesions per 100 kb (Kalinowski et al., 1992; Andrade-Lima et al., 2015; Bohr et al., 1985), we calculated that a single TBL per 100 kb resulted in approximately 20% reduction in transcription, with transcription loss displaying a near-linear relationship at low TBL densities (Figure 2C). However, at higher TBL densities, the data appears to plateau, consistent with the decreasing probability of impacting new genes. To account for this behavior, we fitted a nonlinear third-order polynomial to the data (Figure 2C). As a control, we performed the same experiment in a comparable wildtype primary fibroblast cell line (C5RO) and found that transcription did not decline 24 h after UV, since DNA damage had already been repaired (Supplementary Figures 2C–E), confirming that the transcription decline we observed was indeed due to the induced DNA damage.

To compare these results with our mathematical model predictions, we calculated the percentage of transcription loss per TBL for different doses of DNA damage. We found that the results of the EU stainings and the model match relatively well, with an average absolute error of the model from the data of 7.1%, or 0.95 times the SEM of the experimental data (Figure 2D). Although the model’s predictions differ in accuracy depending on the dose of TBLs (Table 1), it approximates transcription loss reasonably well compared to the experimental data. In summary, our findings illustrate that transcriptional stress induced by a known amount of DNA damage can be modeled with an average absolute error rate of 7.1%, or 0.95 times the experimental SEM within the dose range tested using this method. This is crucial to understand before applying our model to *in vivo* data, as the intrinsic error will be amplified due to biological and technical variation.

**TABLE 1:** Absolute and relative error rates (in % and in #SEMs) of the mathematical model for human fibroblasts as compared to the experimental data based on EU-incorporation levels.

UV dose (J/m^2^)	1	2	3	4	5	6
#TBLs per 100 kb	0.55	1.10	1.65	2.20	2.75	3.30
#TBLs in total	17,600	35,200	52,800	70,400	88,000	105,600
Absolute error of the model (%)	1.18	6.12	0.94	7.05	12.0	15.0
Experimental SEM (%)	5.61	6.11	9.39	9.06	7.86	7.12
Absolute error in #SEMs	0.21	1.00	0.10	0.78	1.53	2.10
Relative error of the model (%)	7.90	29.6	2.76	14.7	20.7	23.0
Relative error in #SEMs	1.41	4.85	0.29	1.63	2.64	3.23

### 2.3 Transcription in aging mouse hepatocytes

Next, we calibrated the model, set out in more detail in (Raseta et al., 2024), for mouse liver aging by sequencing nascent RNA from the livers of young adult mice (N = 3 mice, 15 weeks old) (Supplementary Figures 1C–E; Supplementary Table 2). We used input data from young adult mice to minimize the influence of development and accumulated DNA damage on gene expression. As described above, we assume that the rate at which TBLs accumulate per time unit is constant. We refer to this parameter as rate r . In this case, we specify time in days. Therefore, the total number of TBLs is given by M=rt , where t is the age of the mouse in days. Subsequently, we fitted the transcription loss predicted by the model to experimental data in mouse hepatocytes to estimate the rate of TBL accumulation across the different mouse models, as outlined below. The same equation for transcription loss $\omega_{M}$ was used as previously described for human fibroblasts; however, in this context, the unknown parameter is not $\omega_{M}$ , but M —the total number of TBLs (Figure 1). The expected percentage of transcription loss ( $\left.E\left(\omega_{M}\right)\right)$ differs slightly between mouse liver and human fibroblasts (Figure 2E). To investigate whether this difference could be attributed to gene length, we applied a filtering strategy to both datasets by not only excluding genes shorter than 5 kb, but also those longer than 750 kb. Notably, this additional filtering removed long, relatively highly expressed genes predominantly found in mouse liver (Supplementary Figure S1D), which are largely absent in the human fibroblast dataset (Supplementary Figure S1B). Despite this, the difference in predicted transcription loss between mouse and human remained (Supplementary Figure S1F). After filtering, the number of genes included in the analysis was approximately 9,000 for mouse and 21,500 for human, reflecting a substantial difference in gene count that may also impact the comparison. The lower number of genes detected in mouse liver likely reflects technical differences between the nascent RNA sequencing datasets, including lower sequencing depth, variation in library preparation workflows, and differences in input amount and RNA fragmentation methods. We conclude that the observed variation in transcription loss predictions is driven primarily by the lower number of included genes, with additional contributions from the shorter total genome length in mouse and tissue-specific gene expression differences, rather than by gene length and expression level alone. We quantified nascent transcription levels in mouse liver by administering EU to mice 5 hours prior to euthanasia and subsequently staining the incorporated EU in nascent RNA within paraffin-embedded liver slices. Again, our focus was exclusively on quantifying the EU signal within the nucleoplasm of hepatocytes, i.e., the nucleus without nucleoli. In addition, we filtered out Kupffer cells, because they show an extremely low EU signal compared to hepatocytes. We did this in two steps: first, by applying a size filter on the segmented nuclei (>24.4 µm^2^), and second, by filtering out cells with a very low EU signal (below the blue, horizontal line in Supplementary Figures 4B–E).

An examination of nuclear size in liver slices revealed larger nuclei in aged wildtype mice and mice with DNA repair deficiencies, with a progressive increase observed with advancing age (Supplementary Figures S5A, B). This finding suggests that cells become polyploid, which is a well-known feature of liver aging in wildtype, and in DNA-damage deficient mice (Barnhoorn et al., 2014; Wang et al., 2017). We found a positive correlation between mean EU intensity (per surface unit) and nuclear size (Supplementary Figures 4B–E), which suggests that polyploid cells on average have higher transcription intensity levels compared to diploid cells. Because the mathematical model does not take into account polyploidy, we decided to exclude polyploid nuclei (larger than 70 μm^2^) from our experimental data (to the right of the orange, vertical line in Supplementary Figures 4B–E).

**FIGURE 3:**
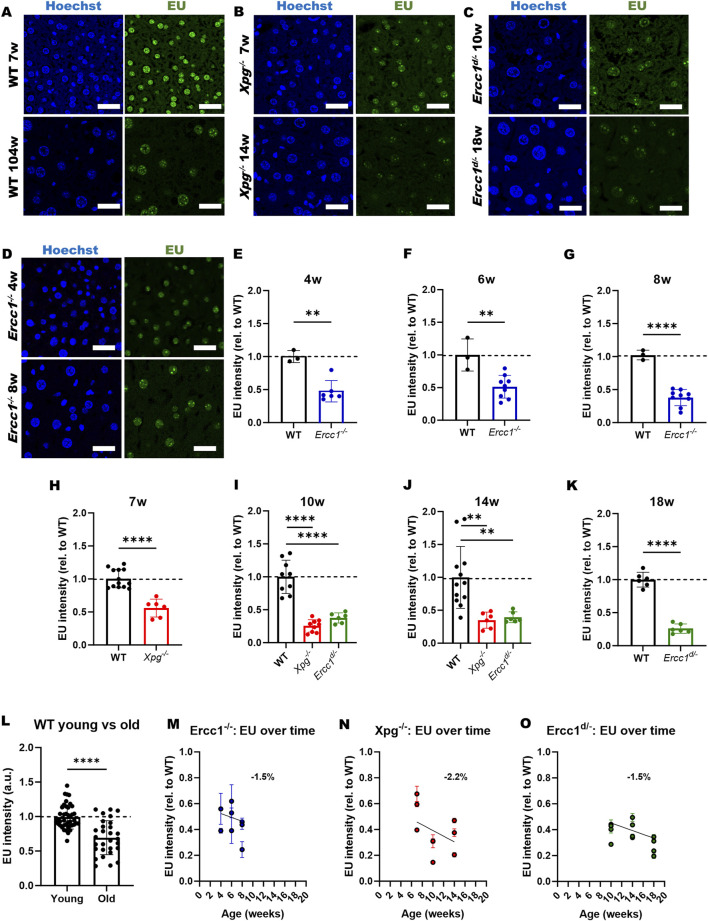
EU incorporation as measure for transcription in mouse liver. **(A–D)** Representative confocal images of mouse liver sections stained for EU and Hoechst. Scale bar = 25 μm. **(A)** Wildtype (WT, 7 and 104 weeks), **(B)** *Xpg* ^*−/−*^ (7 and 14 weeks), **(C)** *Ercc1* ^*d/-*^ (10 and 18 weeks), **(D)** *Ercc1* ^*−/−*^ (4 and 8 weeks). **(E–K)** Average normalized EU intensities in hepatocytes. Data points represent the median of each field of view (FOV); error bars indicate standard deviation (SD) across FOVs. **(E)** 4-week-old WT (N = 1) and *Ercc1* ^*−/−*^ (N = 2), **(F)** 6-week-old WT (N = 1) and *Ercc1* ^*−/−*^ (N = 3), **(G)** 8-week-old WT (N = 1) and *Ercc1* ^*−/−*^ (N = 3), **(H)** 7-week-old WT (N = 2) and *Xpg* ^*−/−*^ (N = 3), **(I)** 10-week-old WT (N = 2), *Xpg* ^*−/−*^ (N = 3), and *Ercc1* ^*d/-*^ (N = 3), **(J)** 14-week-old WT (N = 4), *Xpg* ^*−/−*^ (N = 3), and *Ercc1* ^*d/-*^ (N = 3), **(K)** 18-week-old WT (N = 1) and *Ercc1* ^*d/-*^ (N = 3). **(L)** EU intensities in young (6, 7, 8 weeks, N = 7) and old (104 weeks, N = 4) WT mice, including data from our previous publication (Raseta et al., 2024); bars show medians with 95% CI across FOVs. Unpaired two-tailed Student’s t-test: *p ≤ 0.05; **p ≤ 0.01; ***p ≤ 0.001; ****p ≤ 0.0001. **(M–O)** Normalized EU intensities over time; each data point represents a mouse; error bars indicate SEM across FOVs; slope of linear fit is indicated (%/week). **(M)** *Ercc1* ^*−/−*^ (4, 6, 8 weeks), **(N)** *Xpg* ^*−/−*^ (7, 10, 14 weeks), **(O)** *Ercc1* ^*d/-*^ (10, 14, 18 weeks).

We examined EU levels in hepatocytes from wildtype mice, and from mice with deficiencies in DNA repair, specifically *Ercc1* ^*−/−*^ (Niedernhofer et al., 2006), *Ercc1* ^*d/-*^ (Gregg et al., 2011) and *Xpg* ^*−/−*^ (Barnhoorn et al., 2014) mice. While *Ercc1* ^*−/−*^ and *Xpg* ^*−/−*^ are full knock-out mice, the hypomorphic *Ercc1* ^*d/-*^ variant features a heterozygous seven-amino-acid deletion at the terminus of the CDS, which retains ∼5–10% residual repair activity (Weeda et al., 1997). We found a 30%–50% decrease in DNA repair-deficient livers compared to age-matched wildtype livers (Figures 3A–K). This suggests that progeroid mouse models with DNA repair deficiencies exhibit a similar but more pronounced decline in transcription compared to aged wildtype mice, in a shorter period of time (Figures 3E–L). Additionally, we observed a decline in EU intensity with age in both wildtype mice (Figures 3A,L) and mice with DNA repair deficiencies (Figures 3B–D,M–O). Specifically, there was a 25% decrease in EU intensity between 7-week-old wildtype livers and 2-year-old wildtype livers (Figure 3L), and an average decrease of 1.7% per week in DNA repair-deficient mice (compared to a weekly 0.3%-decrease in wildtype mice), although the data showed considerable variability (Figures 3M–O).

**FIGURE 4:**
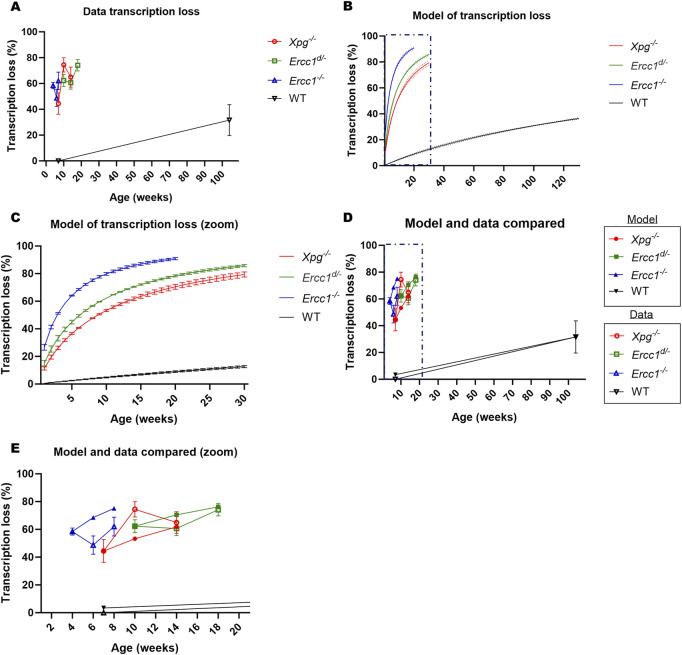
Mathematical model on DNA damage and transcription loss. **(A)** Average percentage of transcription loss in hepatocytes, as measured by EU staining of liver sections for each mouse model (wildtype, *Xpg* ^*−/−*^, *Ercc1* ^*d/-*^ and *Ercc1* ^*−/−*^) at different, relevant ages. Error bars represent SEM across mice (N = 3 for *Xpg* ^*−/−*^, *Ercc1* ^*−/−*^ and *Ercc1* ^*d/-*^ mice, N = 4 for wildtype mice). **(B)** Percentage of transcription loss in hepatocytes, as predicted by the mathematical model for each mouse model (wildtype, *Xpg* ^*−/−*^, *Ercc1* ^*d/-*^ and *Ercc1* ^*−/−*^), across different, relevant ages. Error bars represent SD based on nascent RNA sequencing data of three independent mice. **(C)** As in **(B)**, but for a narrower age range. **(D)** Comparison of average transcription loss between predictions from our mathematical model and experimental data from liver sections. Error bars represent the standard deviation (SD) for the model and the standard error of the mean (SEM) for the experimental data. **(E)** As in **(D)** but for a narrower age range.

We estimated the rate r at which TBLs accumulate in each mouse model (Table 2) by performing a goodness-of- fit analysis on the first EU incorporation data point (Figure 4A). The second and third data point were used to validate the predictive performance of the calibrated model and calculate the error rate. For wildtype mice, we fitted the model to the last data point, and then conversely predicted the first data point to estimate the error rate, since there were only two data points, and the first (at 7 weeks) was assumed to have 0% transcription loss. Using the estimated rates, we calculated transcription loss for each different mouse model (Figures 4B,C). We used the closed-form formula, described above (Figure 1), for the expected value of $\omega_{M}$ : $E(\omega_{\left.M\;\right)}=\sum_{j=1}^{2N}\alpha_{j}\left(1-{\left(1-q_{j}\right)}^{M}\right)$ , as derived in Proposition 2 of (Raseta et al., 2024), where M=rt . The transcription loss predicted by the model aligns reasonably well with the observed transcription loss in each mouse model (Figures 4D,E). We calculated both the absolute and relative error rates for each second and third data point, and found that the largest errors occurred at the second data points (Table 2), probably due to high biological variability in the experimental data.

**TABLE 2:** Occurrence rate r of unrepaired TBLs in each mouse model, including standard deviation (SD), absolute and relative error compared to experimental data (of the second and third data point and the average) in %, and 50% survival age in weeks.

Genotype	Number of unrepaired TBLs per day ±SD	Absolute error $\omega_{M\;}$ model (%)			Relative error $\omega_{M\;}$ model (%)			50%-survival age (weeks)
		2nd point	3rd point	Average	2nd point	3rd point	Average	
*Xpg* ^*−/−*^	1,621 ± 150	21.6	3.9	12.7	29.9	6.0	17.5	18.2
*Ercc1* ^*d/-*^	2,315 ± 163	9.7	1.7	5.7	16.0	2.2	9.1	21.8
*Ercc1* ^*−/−*^	4,978 ± 372	19.8	13.1	16.4	40.6	21.1	30.1	8.0
Wildtype	62 ± 7.0	NA	NA	3.6	NA	NA	NA	120

Among the mouse models investigated, the *Ercc1* ^*−/−*^ mutant exhibited the most severe age-dependent decline in EU incorporation, showing approximately 50% transcription loss at 4 weeks (Figure 3E), which is consistent with previous research (Gregg et al., 2011). In line with the *Xpg* ^*−/−*^ and *Ercc1* ^*d/-*^ mouse models generally displaying milder age-related phenotypes and longer lifespans (Niedernhofer et al., 2006; Barnhoorn et al., 2014), transcriptional activity declined by approximately 50% at around 7 and 10 weeks of age, respectively (Figures 3H,I). This aligns with their longer survival and the lower predicted daily accumulation of TBLs (Table 2).

**FIGURE 5:**
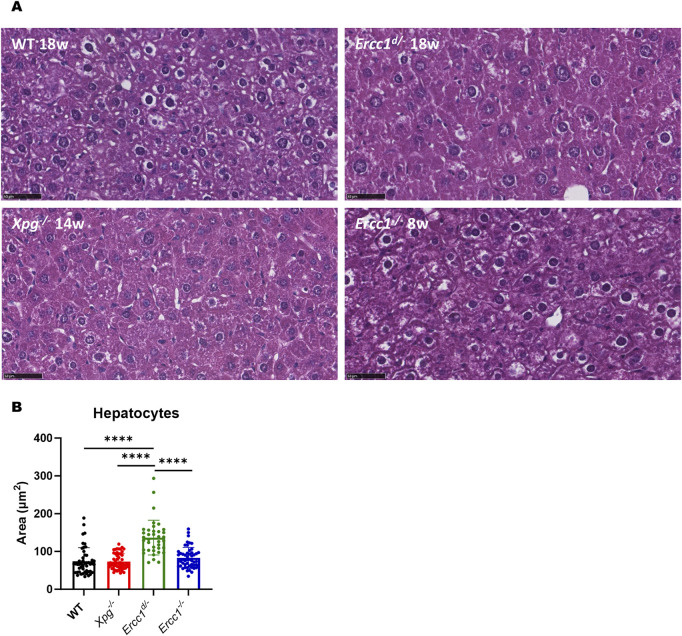
Liver pathology. **(A)** Representative images of H&E staining of liver: WT 18 weeks old (upper left panel), *Ercc1* ^*d/-*^ 18 weeks old (upper right panel), *Xpg* ^*−/−*^ 14 weeks old (lower left panel) and *Ercc1* ^*−/−*^ 8 weeks old (lower right panel), all female. Magnification: ×40. Scale bar: 50 µm. **(B)** Area of hepatocyte nuclei in wildtype, *Xpg* ^*−/−*^, *Ercc1* ^*d/-*^ and *Ercc1* ^*−/−*^ mice, as measured using H&E staining. Unpaired two-tailed Student’s t-test: *p ≤ 0.05; **p ≤ 0.01; ***p ≤ 0.001; ****p ≤ 0.0001.

The difference between the *Xpg* ^*−/−*^ and *Ercc1* ^*d/-*^ mouse models, however, was relatively small. Although their lifespans are comparable, with *Xpg* ^−/−^ mice exhibiting a slightly shorter lifespan, the model predicted a higher daily number of TBLs in *Ercc1* ^d/-^ livers than in *Xpg* ^−/−^ livers, contrary to what their lifespans suggest. Supporting the model’s predictions, we found more severe liver pathology in *Ercc1* ^*d/-*^ mice than in *Xpg* ^*−/−*^ mice (Figure 5A). In particular, we detected more polyploidy in *Ercc1* ^*d/-*^ livers compared to *Xpg* ^*−/−*^ or wildtype livers (Figure 5B; Supplementary Figure S5A). Thus, although *Xpg* ^*−/−*^ mice have a shorter lifespan, they may accumulate TBLs in hepatocytes at a lower rate than *Ercc1* ^*d/-*^ mice. This discrepancy may reflect survivorship bias, meaning that the observed lifespan reflects only the organs most affected (e.g., brain), while other organs like the liver may accumulate differing levels of TBLs without causing early death. Notably, neither model dies from liver pathology, as seen in *Ercc1* ^−/−^ mice (Niedernhofer et al., 2006), but instead develop fatal neurological impairments (Gregg et al., 2011; Barnhoorn et al., 2014).

To further explore the impact of DNA repair deficiency, we assumed that *Ercc1* ^*−/−*^ mice are unable to repair any type of transcription-blocking DNA damage, while *Xpg* ^*−/−*^ mice retain the ability to repair crosslinks. *Ercc1* plays an essential role not only in NER, but also in crosslink repair through the Fanconi Anemia (FA) pathway (Friboulet et al., 2013), whereas *Xpg* is essential for NER but does not participate in crosslink repair (De Silva et al., 2002). Although DSBs are TBLs, their low frequency, high toxicity, and functional repair in both *Ercc1* ^*−/−*^ and *Xpg* ^*−/−*^ cells render their effect on transcription negligible. Therefore, the difference in interstrand crosslink repair capacity is most likely the primary factor contributing to the higher daily accumulation of TBLs and the increased polyploidy observed in *Ercc1* ^*−/−*^ compared to *Xpg* ^*−/−*^ mice. Based on this, we estimated that *Xpg* ^*−/−*^ mice repair approximately 3,357 interstrand crosslinks per day $\left(4978-1621=3357\right)$ , which corresponds to approximately 67% of the total TBLs in *Ercc1* ^*−/−*^ mice $\left(\frac{3357}{4978}*100=67.4\%\right.$ ) (Table 2). This suggests that *Xpg* ^*−/−*^ mice retain a TBL repair capacity of 67.4%. However, given the high cytotoxicity of interstrand crosslinks, this number appears unexpectedly high and should be interpreted with caution. Assuming wildtype mice accumulate TBLs at the same rate as *Ercc1* ^*−/−*^ mice, but repair the majority of it, we estimated that approximately 1.2% of lesions persist daily ( $\frac{62}{4978}=0.012=1.2\%$ ), accumulating with age, while roughly 98.8% of DNA lesions are repaired ( 100−1.2=98.8% ) (Table 2). However, it is unlikely that specific lesions persist indefinitely in wildtype mice. A more plausible explanation is that TCR becomes progressively slower with age, leading to gradual transcriptional stress, especially in long genes.

*Ercc1* ^*d/-*^ mice are predicted to retain approximately 54% of TBL repair capacity $\left(\frac{4978-2315}{4978}*100=53.5\%\right)$ (Table 2). These mice have one truncated *Ercc1* allele and one null allele, resulting in an RNA transcript level of the truncated allele that accounts for approximately 7.5% of total *Ercc1* expression in wildtype mice (Weeda et al., 1997). The truncated version of the ERCC1 protein lacks seven amino acids in the C-terminal HhH_2_ domain, which normally dimerizes with XPF and may bind to DNA (Weeda et al., 1997). As a result, the DNA repair function of the truncated ERCC1 protein is likely reduced compared to the wildtype version, though this has not been quantified. Nevertheless, assuming that *Ercc1* ^*d/-*^ mice retain 7.5% of the repair capacity of wildtype mice, they are predicted to accumulate approximately 4,605 TBLs daily $\left(4978-0.075*4978=4605\right)$ . This suggests that our model’s estimate of 2,315 TBLs per day (Table 2) may be on the low side.

## 3 Discussion

In this study, we attempted to quantify the accumulation of transcription-blocking DNA damage during aging by integrating global transcription level measurements with a mathematical model based on fundamental biological assumptions (Raseta et al., 2024). By integrating *in vitro* experimental data with our model, we estimated its absolute error rate in a controlled environment to be 7.1%. Using *in vivo* experimental data, we estimated the daily incidence of DNA lesions in hepatocytes across various mouse models. Wildtype mice were found to accumulate approximately 62 TBLs daily, whereas DNA repair-deficient mice exhibited a markedly higher burden, accumulating 1,600–5,000 TBLs daily.

Our findings are largely consistent with those reported in previous studies. Previous research, using mass spectrometry, discovered approximately 132,500 cyclopurine lesions in the *Ercc1* ^*d/-*^ liver genome at 18 weeks, or 5.0 cyclopurines per 100 kb of the diploid mouse genome (Robinson et al., 2018), suggesting that approximately 68% of TBLs are cyclopurines. This is close to our findings of 193,450 TBLs (or 7.3 TBLs per 100 kb) in 18-week old *Ercc1* ^*d/-*^ mice. These estimations suggest that cyclopurines, caused by oxidative stress and alkylating agents, are a major type of DNA lesion in these mice. Another study quantified the number of cyclopurine lesions in old wildtype livers using an antibody-based approach and found that 24-month-old mice had approximately 2.4 cyclopurine lesions per 100 kb (Mori et al., 2019), corresponding to approximately 40% transcription loss as predicted by our model. In our study, 24-month-old wildtype mice exhibited a 30.5% loss in transcription, which our mathematical model predicted to be caused by approximately 1.58 TBLs per 100 kb. Since this estimate is lower than the number of cyclopurine lesions specifically, our findings further support the conclusion that cyclopurines are a predominant type of TBL in the liver.

In addition to cyclopurines, interstrand crosslinks are potentially of significant interest in the context of transcriptional stress. Endogenous interstrand crosslinks are formed by for example, aldehydes, nitric oxide or oxidized abasic lesions (Clauson et al., 2013; Housh et al., 2021). Since aldehydes are formed during lipid and alcohol metabolism, dietary habits can influence the formation of DNA lesions such as interstrand crosslinks (Clauson et al., 2013; Housh et al., 2021). Nitric oxide, a signaling molecule involved in vasoregulation, has DNA crosslinking capabilities and is a by-product of nitrous acid. Nitrous acid, in turn, is produced from nitrates in processed meat, further linking crosslinks to dietary choices (Clauson et al., 2013; Housh et al., 2021). Additionally, oxidized abasic sites—abundant lesions arising from the spontaneous hydrolysis of DNA—can form interstrand crosslinks when located on opposing strands in close proximity. According to our model’s predictions (Table 2), we hypothesized that approximately 67% of TBLs in *Ercc1* ^*−/−*^ mice are interstrand crosslinks. This suggests that, in a TBL repair-deficient model, 67% of transcriptional stress is caused by interstrand crosslinks, underscoring the significance of this type of DNA damage in causing transcriptional stress.

DNA-associated advanced glycation end products (DNA-AGEs), alongside cyclopurines and interstrand crosslinks, could play an important role in the context of transcriptional stress. DNA-AGEs form through non-enzymatic reactions between reducing sugars or reactive carbonyl species (e.g., methylglyoxal) and DNA bases, resulting in irreversible modifications. These reactions are mostly driven by metabolic processes such as glycolysis, oxidative stress, and lipid peroxidation, and are strongly correlated to life style and dietary choices (Chaudhuri et al., 2018). DNA-AGEs are especially prevalent in hyperglycemic conditions (Jaramillo et al., 2017). A previous study quantified a specific and relatively stable type of AGEs called carboxyethyl-deoxyguanosine (CEdG) in wildtype mouse livers using mass spectrometry (Jaramillo et al., 2017). Approximately one CEdG was observed per 10^6^ deoxyguanosines (dGs) in genomic DNA from 28- to 36-week-old livers, corresponding to an estimated 1,113 CEdGs per genome. According to our model, this lesion load corresponds to approximately 1.7% transcription loss, suggesting that AGEs may contribute modestly to transcriptional stress.

Working with a simplified mathematical model inevitably introduces uncertainty, as illustrated by the model’s error rate for human fibroblasts following UV-induced damage (Table 1). Moreover, the *in vitro* experimental data from human fibroblasts appear to follow a more linear trend than the model predicts, with the model plateauing at a lower number of TBLs. We speculate that these differences between experimental data and model may be caused by several factors. Importantly, both the experimental data and the model exclude ribosomal genes: ribosomal RNA is filtered out in the experimental data by excluding nucleolar staining, and ribosomal genes are also removed during the mapping of nascent RNA sequencing input data.

One factor that could contribute to the discrepancy is that the model assumes transcription ceases entirely upon the acquisition of a lesion anywhere in the transcribed strand of a gene. In contrast, in cells, RNA polymerases continue transcription up to the lesion. Furthermore, in the model, damage accumulates gradually, whereas UV-induced damage in fibroblasts occurs within seconds. Although fibroblasts are given 24 h to adapt to the damage, certain factors such as the abrupt imbalance in RNA polymerase II turnover, may have persistent effects. In addition, DNA damage is exposure-dependent (e.g., UV in sunlight), and with aging, most cellular processes—including protective systems such as antioxidant defenses and DNA repair mechanisms—decline in function. This functional decline likely increases the rate at which DNA damage accumulates with age. However, for simplicity, our model assumes a constant rate of DNA damage accumulation.

A further limitation is that the model does not account for DNA replication, which can displace stalled RNA polymerases and thereby restore lesion accessibility for the repair machinery. Similarly, polyploidization could mitigate transcriptional blockages by providing additional, undamaged copies of the genome for transcription. The model also omits the potential bypass of TBLs via transcriptional bypass, a process in which RNA polymerases transiently bypass DNA damage to allow continued transcription (Walmacq et al., 2015). Another consideration is the use of nascent RNA imaging with EU labeling, which may preferentially capture longer transcripts. Shorter RNAs are transcribed, processed, and exported or degraded more rapidly, potentially reducing the window for EU incorporation and detection. As such, these transcripts might be underrepresented, although this likely depends on labeling duration and transcript-specific kinetics. Furthermore, it is worth noting that the model does not consider the possibility of damage occurring in the gene encoding RNAPII itself or other factors important for transcription and TC-NER. Such an event could lead to reduced production of these components and could disproportionately affect global transcription. Together, these factors highlight the limitations of the mathematical model in fully capturing the dynamic and complex biological responses of cells to TBLs.

Finally, while human dermal fibroblasts and mouse hepatocytes serve as a solid starting point for investigation, it is essential to acknowledge that our focus on this specific cell type does not fully represent the dynamics of transcriptional stress and aging across an entire organism. Therefore, caution is required when extrapolating these results to the broader spectrum of organismal aging. Expanding the study to include additional mouse tissues, such as mouse dermal fibroblasts, could offer valuable insights. Another limitation of our mouse study was the restricted number of time points and the small sample size per time point, which introduced considerable variability between individual mice. A further limitation of our study is the strong dependence of the model’s predictions on the nascent RNA-seq datasets used as input. Variations in experimental protocols, sequencing depth, RNA quality, and data processing can substantially influence the number and identity of detected genes, thereby affecting transcription loss estimates. This dependence may impact the generalizability of our findings across different tissues and experimental conditions. Consequently, the findings reported here should be taken with caution due to numerous unknowns and inherent limitations. Nevertheless, as a first attempt to quantify transcriptional stress in these models, this study provides novel and important insights for the field.

In summary, our mathematical model advances the understanding of DNA damage accumulation and its implications for transcriptional stress and aging. By predicting outcomes such as the daily persistence of approximately 15 unrepaired TBLs in the liver of wildtype mice, the model offers valuable insights into the accumulation of DNA damage over time, contributing to a deeper understanding of the aging process. Interestingly, in combination with previous studies (Robinson et al., 2018; Mori et al., 2019) and experimental data, our model suggests that cyclopurines could be one of the dominant types of DNA damage in aging. Future research should aim to validate this finding and identify other specific DNA lesions, beyond cyclopurines, that significantly contribute to transcriptional decline in aged tissues. Moreover, studies of methods for reducing TBL accumulation could potentially yield promising strategies to mitigate the aging process.

## 4 Materials and methods

### 4.1 Mice

Mouse housing and experiments were conducted in strict accordance with the Animal Welfare Act of the Dutch government, following the Guide for the Care and Use of Laboratory Animals and adhering to guidelines approved by the Dutch Ethical Committee in full compliance with European legislation. The institutional ethical committee for animal care and usage granted approval for the animal protocol.

DNA repair-deficient premature aging mouse models, generated in-house, and their wildtype littermates in F1 C57BL6J/FVB (1:1) hybrid background were euthanized using cervical dislocation at 10, 14, and 18 weeks for *Ercc1* ^*d/−*^ mutants (Niedernhofer et al., 2006), and 7, 10, and 14 weeks for *Xpg* ^*−/−*^ mutants (Barnhoorn et al., 2014). Wildtype littermates were euthanized at 4, 6, 7, 8, 10, 14, 18 weeks, and 104 weeks of age. Euthanasia was performed to collect tissues at predetermined ages relevant to disease progression, while minimizing animal suffering. All procedures, including euthanasia, were approved by the institutional ethical committee and conducted in full compliance with Dutch and European legislation. The wildtype C57BL6J and FVB strains, commonly sourced from the Jackson Laboratories, were used to maintain a standardized genetic background.

All animals were bred and kept on AIN93G synthetic pellets (Research Diet Services; gross energy content 4.9 kcal g−1 dry mass, digestible energy 3.97 kcal g−1). They were housed in a controlled environment (20–22 °C, 12 h light:12 h dark cycle) and individually accommodated in ventilated cages under specific pathogen-free conditions at the Animal Resource Center (Erasmus University Medical Center). Statistical methods were employed to determine sample sizes, resulting in a group size of 3 animals, only males. Data collection and analysis were not performed blind to the experiment conditions, and randomization of animals to experimental groups was not applicable.

### 4.2 Nascent RNA labeling *in vivo*

Mice were intraperitoneally injected with 5-EU (AXXORA) at a dosage of 0.088 mg per gram of body weight. Five hours post-injection, mice were euthanized, and tissue samples were collected, formalin-fixed and embedded in paraffin.

### 4.3 Cell culture

XP25RO cells (XPA knock-out primary patient skin fibroblasts) were cultured at 37 °C in DMEM with 10% FBS and 1% PS at 5% CO_2_ and 20% O_2_, passaged weekly at a 1:7 ratio. C5RO cells (wildtype primary skin fibroblasts) were cultured in F10 with 15% FBS and 1% PS, under the same conditions. The XP25RO and C5RO cell lines present in this study were a kind gift from Arjan Theil. Cells were seeded in 35 mm dishes with glass slides, grown to confluence, and maintained for 2 weeks with medium refreshed bi-weekly. Subsequently, cells were exposed to UV-C radiation at doses of 0, 1, 2, 3, 4, 5 or 6 J/m^2^ using a 254-nm germicidal lamp (Philips). After a 24-h period, cells were fixed using 2% PFA for 15 min, after incubation with EU (5-ethynyluridine, 0.325 mM, 1 h) or EdU (5-ethynyl-2-deoxyuridine, 10 μM, 2 h) to label newly synthesized RNA or DNA, respectively.

### 4.4 EU or EdU labeling on human cells

Following fixation, cells were permeabilized with 0.1% Triton X-100 in PBS for 10 min at room temperature. A click-it cocktail was prepared per 24-mm coverslip using 79 μL 50 mM Tris (pH 7.0), 1 μL Atto 488 azide (Lumiprobe, 11,830), 10 μL 40 mM CuSO_4_·5H_2_O, and 10 μL 100 mM ascorbic acid. After PBS washes, 100 μL of the cocktail was added, and cells were incubated for 1 h at room temperature in the dark. Cells were then washed (3× PBS-T, 1× PBS), stained with Hoechst 33,342 (ThermoFisher, 62,249; 20 μM, 20 min), and mounted in Aqua Poly mount (Polysciences, 18,606–20). Slides were dried overnight in the dark and imaged using a Zeiss LSM700 confocal microscope with a 20× dry lens.

Images were analyzed using an ImageJ macro that segmented nuclei based on Hoechst staining with Otsu thresholding. Nuclei between 24.4 and 250 μm^2^ and circularity >0.6 were selected to focus on diploid hepatocytes. Nucleoli were segmented within nuclei using a ‘Moments’ threshold after background subtraction. The macro then measured mean nucleoplasmic signal in the original EU channel by subtracting nucleoli from nuclei. Nuclear area was also quantified. A Python script calculated mean EU intensity in nucleoplasm, and data from four independent experiments, normalized to 0 J/m^2^ controls, were graphed in GraphPad Prism. EdU-positive cells were defined by a manually set intensity threshold per experiment.

Our study concentrated on quantifying the EU signal within the nucleoplasm of cells, excluding the nucleoli (Supplementary Figure S2B). This approach was chosen because rRNA transcription in the nucleoli typically remains high, likely due to the fact that rRNA genes are relatively short, present in >100 copies and intensively transcribed, which renders them less prone to DNA damage (Eickbush and Eickbush, 2007). Additionally, since the mathematical model does not take into account polyploidy, we filtered out polyploid nuclei (nuclei larger than 250 μm^2^), based on the nuclei segmentation in the Hoechst channel (Supplementary Figure S4A).

### 4.5 Nascent RNA sequencing on human fibroblasts

For nascent RNA sequencing, TERT-immortalized C3RO cells (wildtype skin fibroblasts) were grown in DMEM with 10% FBS and 1% PS at 5% CO_2_ and 20% O_2_ until fully confluent, for steady-state labeling. Cells were mock-treated with DMSO for 48 h, followed by a 15-min pulse with 1 mM EU at 37 °C. Four independent biological replicates were performed. Total EU-labeled RNA was then extracted using the Qiagen miRNeasy kit (217,004), according the manufacturer’s protocol.

Nascent RNA was isolated using the Click-iT Nascent RNA Capture Kit (ThermoFisher, C10365) according to the manufacturer’s guidelines, with the largest recommended input amounts. EU RNA-bound beads were suspended in fragment-, prime-, and elute buffer from the KAPA RNA HyperPrep Kit (Roche, KK8540), heated to 94 °C for 6 min, then prepared into libraries with 15 PCR cycles and purified. Library quality was assessed using the High Sensitivity D1000 assay on a TapeStation system (Agilent). Libraries were sequenced using a NovaSeq 6000 system (Agilent), with equal RNA input per sample.

The EU-sequencing reads were preprocessed using FastQC v.0.11.9, FastQScreen v.0.14.0, and Trimmomatic v.0.35 (Bolger et al., 2014), and aligned to human ribosomal DNA, mitochondrial sequences (UCSC (Nassar et al., 2023), hg38), and the human reference genome (GRCm38) using Tophat2 v.2.0.9 (Trapnell et al., 2009), with default settings, except for the -g1 option. Non-expressed genes (0 RPM) were excluded. For Figure 4A and Supplementary Figure S1, genes shorter than 5 kb were excluded to match mouse hepatocyte comparisons; no length filtering was applied in other analyses.

### 4.6 EU labeling

Male mouse liver tissues were sectioned into 3 μm slices using a microtome (HM335E, Microm). After deparaffinization and rehydration, slices were washed with PBS and permeabilized with 0.1% Triton X-100 in PBS for 10 min at room temperature. A click-it cocktail was prepared by mixing 79% 50 mM Tris buffer (pH 7.0), 1% Atto 488 azide (Lumiprobe, 11,830), 10% 40 mM CuSO4·5H2O, and 10% 100 mM ascorbic acid. Following permeabilization, slices were washed, incubated with 150 µL click-it cocktail for 30 min in the dark at room temperature, washed again with PBS, then stained with Hoechst 33,342 (Invitrogen, C10337) for 15 min and washed. Slices were mounted using Aqua Poly mount (Polysciences, 18,606–20) and dried overnight in the dark. Imaging was performed with a Zeiss LSM700 confocal microscope using a 20x dry lens.

Images were analyzed using an ImageJ macro that segmented nuclei based on Hoechst staining with Otsu thresholding. Nuclei sized between 24.4 μm^2^ and 70 μm^2^ with circularity >0.7 were selected to focus on diploid hepatocytes. Nucleoli were segmented using a ‘Moments’ threshold after background subtraction. The macro measured the mean nucleoplasmic signal by subtracting nucleoli from nuclei on the EU channel (Supplementary Figure S2B), and nuclear area was quantified. A Python script calculated the median mean EU intensity per field of view. Graphs were generated with GraphPad Prism. Data from *Ercc1* ^*d/-*^ *, Ercc1* ^*−/−*^ *,* and *Xpg* ^*−/−*^ livers were normalized per imaging session using a wildtype 7-week reference sample, and further normalized within age groups by dividing by corresponding wildtype values.

### 4.7 HE staining

Female mouse liver tissues were sectioned into 3 μm slices (HM335E, Microm). After deparaffinization and rehydration, slices were stained with Gill’s No. 3 hematoxylin (Merck, GHS332) for 5 min, rinsed in running tap water for 1 min, and briefly in 70% ethanol. Tissues were then counterstained with Eosin-Y (Sigma, 230,251) for 30 s, followed by dehydration through graded ethanol series (70%, 95%, 100% twice) and xylene (twice). Slides were mounted with Pertex (00811-EX) and scanned on a Nanozoomer (Hamamatsu). Hepatocyte nuclear area was analyzed using NDP View 2 software.

### 4.8 Nascent RNA sequencing (mouse liver)

Nascent RNA sequencing of young mouse liver was performed as described elsewhere (Gyenis et al., 2023). In short, total RNA was extracted from snap-frozen liver using the miRNeasy kit (QIAGEN) with on-column DNase treatment. RNA quality was confirmed by Bioanalyzer (RIN >8.0). EU-labeled nascent RNA was isolated using the Click-iT Nascent RNA Capture Kit (Thermo Fisher Scientific) via biotin azide Click chemistry and purified with MyOne Streptavidin T1 beads. On-bead cDNA synthesis was done with SuperScript II and random hexamers, followed by end repair, A-tailing, adapter ligation, and 15 PCR cycles using the TruSeq mRNA Sample Preparation Kit v2 (Illumina). Libraries were purified, quantified (Bioanalyzer DNA1000), pooled equally, and sequenced three per lane on a HiSeq 2,500.

EU-seq reads were preprocessed using FastQC v.0.11.9 and Trimmomatic v.0.35 (Bolger et al., 2014), using the parameters: SLIDINGWINDOW:4:15 LEADING:3 TRAILING:3 ILLUMINACLIP:adapter.fa:2:30:10 LEADING:3 TRAILING:3 MINLEN:36. Following preprocessing, the reads were sequentially aligned to mouse ribosomal DNA (BK000964.3), mitochondrial sequences (UCSC, mm10), and the mouse reference genome (GRCm38/mm10) using Tophat2 v2.0.9 (Trapnell et al., 2009), with default settings, except for the -g1 option. All genes, exons, and introns from RefSeq (release: 95) were retrieved from the UCSC Genome Browser (Nassar et al., 2023). The gene lists were then consolidated to the longest transcript per gene. Genes shorter than 5 kb in length were excluded. The nascent expressed gene set comprises genes with at least one read mapping to an intronic region in any of the three young samples.

### 4.9 Determining the timepoint of acute transcriptional response resolution (24h)

A previous study utilizing bromouridine-tagged nascent RNA sequencing (Bru-Seq) (Andrade-Lima et al., 2015) was used to identify the timepoint at which the acute transcriptional response is resolved. The study employed wildtype HF1 (hTERT-immortalized), XPC-deficient XP67TMA (primary), and CSB-deficient CS1AN (primary) human fibroblasts, which were irradiated with 10 J/m^2^ UV and subjected to Bru-Seq at 0.5, 2, 6, and 24 h post-treatment. As a control, untreated fibroblasts were subjected to the same sequencing protocol.

We quantified the intragenic distribution of reads using a metric called tilt, defined as the difference in the slopes of linear regressions fitted to RPKM values along the gene body before and after UV-induced DNA damage. A negative tilt indicates reduced transcription toward the 3′end, reflecting impaired transcription elongation due to transcription-blocking lesions (TBLs). Shortly after UV exposure, reads accumulate at the 5′end of genes, as TBLs hinder RNA polymerase progression. In wildtype and GG-NER-deficient cells, this distribution normalizes within 24 h through TC-NER. In contrast, TC-NER-deficient CSB^−/-^ cells fail to restore normal distribution, as stalled RNA polymerases are not removed and block access to other repair pathways. Although overall transcription levels seem to recover, transcript completion remains impaired. As 5′transcription levels increase and spread toward the 3′end, the persistent stalling leads to incomplete transcripts, explaining why tilt recovery lags behind average transcription recovery.

### 4.10 Fitting the model

The daily rate of DNA damage was determined *in silico* by minimizing the error between the model from (Raseta et al., 2024) and experimentally observed transcriptional loss. For each mouse in the nascent RNA dataset, the model was calibrated to find the daily number of transcription-blocking lesions (TBLs) that best predicted transcription loss at the first time point (4 weeks for *Ercc1* ^*−/−*^, 7 weeks for *Xpg* ^*−/−*^ and 10 weeks for *Ercc1* ^*d/-*^ mice). Predictions were then tested against the second and third data points. For wildtype mice, only two data points were available; since no transcription loss was observed at 7 weeks, the model was instead calibrated using the second time point (104 weeks) to avoid underestimating damage, then applied to predict the first time point. Relative errors were calculated by comparing model predictions to experimental data at the validation time points, with formulas provided for relative and absolute errors (%) per time point.

Relative error was calculated by comparing the model’s predicted average transcription loss to experimental data at the second and third time points, since the first time point was used for fitting. For wildtype mice, only the first time point’s error was considered, as the second was used for fitting. The formulas for relative and absolute error (%) per time point are as follows:

$$\text{relative error}\left.=\right|\frac{\omega_{M,model-}\omega_{data}}{\omega_{data}}\left.*100\%\right|$$

$$a\text{bsolute error}=\left|\omega_{M,model-}\omega_{data}\right|$$

where $\omega_{M,model}$ is the expected transcription loss predicted by the model (upon M accumulated TBLs which, due to constant accumulation rate, has a direct correspondence to time measured in days), and $\omega_{data}$ is the average transcription loss measured experimentally.

## Data Availability

The original contributions presented in the study are publicly available. Human fibroblast nascent RNA sequencing data (C3RO cells, n = 4) have been deposited under BioProject accession number PRJNA1017406 (NCBI SRA accessions: SRR26060338, SRR26060336, SRR26060309, SRR26060320). Mouse liver EU-seq data (n = 3) are available under NCBI SRA accession number PRJNA603447 (SRX7638577, SRX7638578, SRX7638579).
