# Supplementary material for: Transcriptional stress in aging: integrating experimental data and modeling to quantify DNA damage accumulation
Source: Front Mol Biosci. 2025 Sep 8;12:1659589. doi: 10.3389/fmolb.2025.1659589 (PMC12450475; doi:10.3389/fmolb.2025.1659589)
Supplement: Supplementary file 2 [file Supplementaryfile1.docx]

Supplementary Material

# Supplementary Figures and Tables

## Supplementary Figures


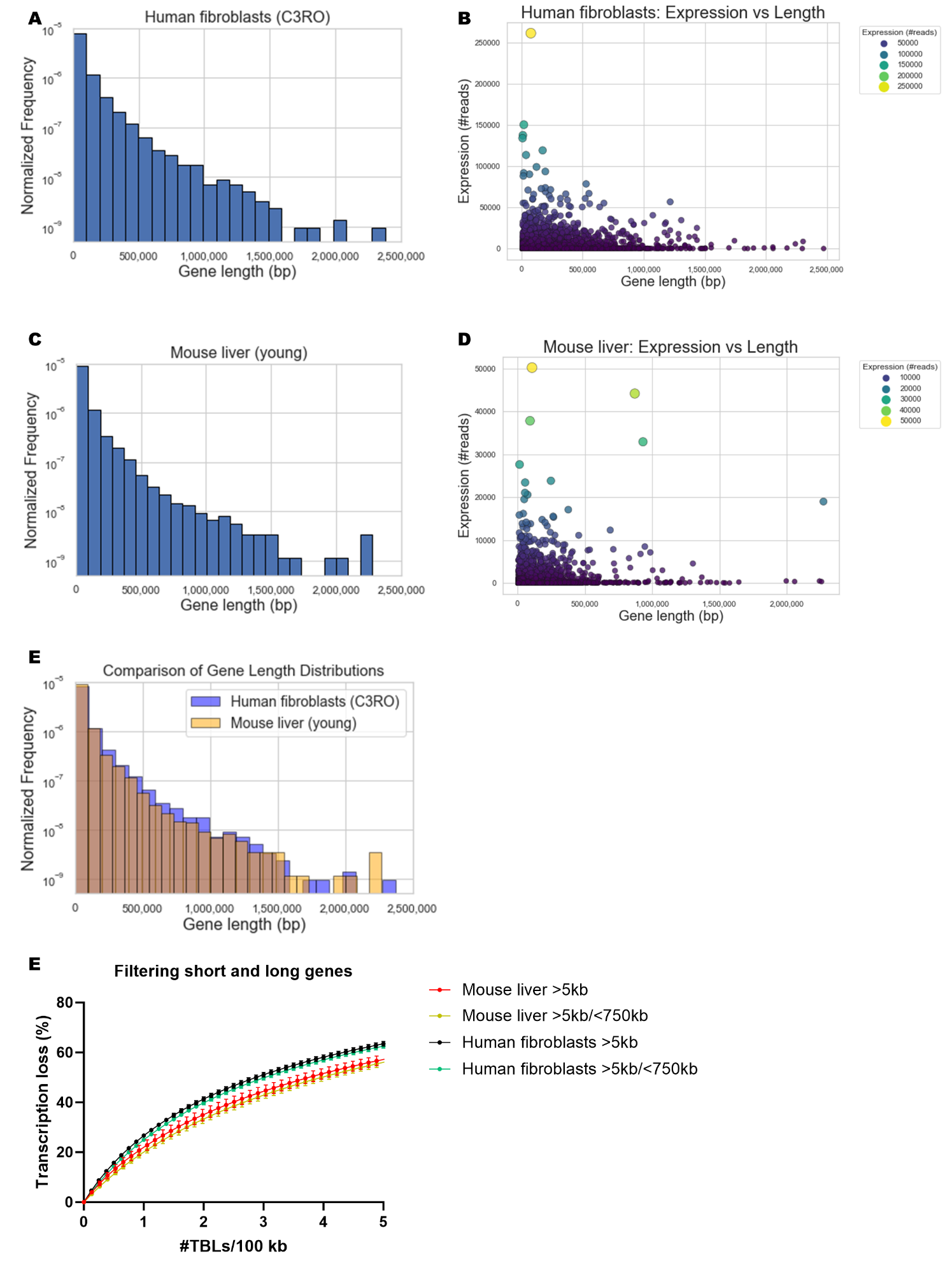


**Supplemental Figure 1: Input expression data from nascent RNA sequencing.** **(A)** Normalized logarithmic histogram of gene lengths (bp) in human fibroblast (C3RO) nascent RNA-seq. **(B)** Scatterplot of expression (read count) versus gene length (bp) for human fibroblasts. Each dot represents one gene (N = 21,636). **(C)** Normalized logarithmic histogram of gene lengths (bp) in young mouse liver nascent RNA-seq. **(D)** Scatterplot of expression (read count) versus gene length (bp) for young mouse liver. Each dot represents one gene (N = 9,661). **(E)** Comparison of normalized logarithmic histograms of gene lengths (bp) between human fibroblast and young mouse liver datasets. **(F)** Transcription loss $E(\omega_{M})$ as a function of the number of transcription-blocking lesions (TBLs) M per 100 kb, as predicted by the mathematical model for mouse liver and human fibroblasts. In both datasets, genes shorter than 5 kb were excluded. Results are shown both without and with an additional filter excluding genes longer than 750 kb (N = 21,451 genes for human fibroblasts and N = 9000 genes for mouse liver). Error bars indicate the standard deviation.


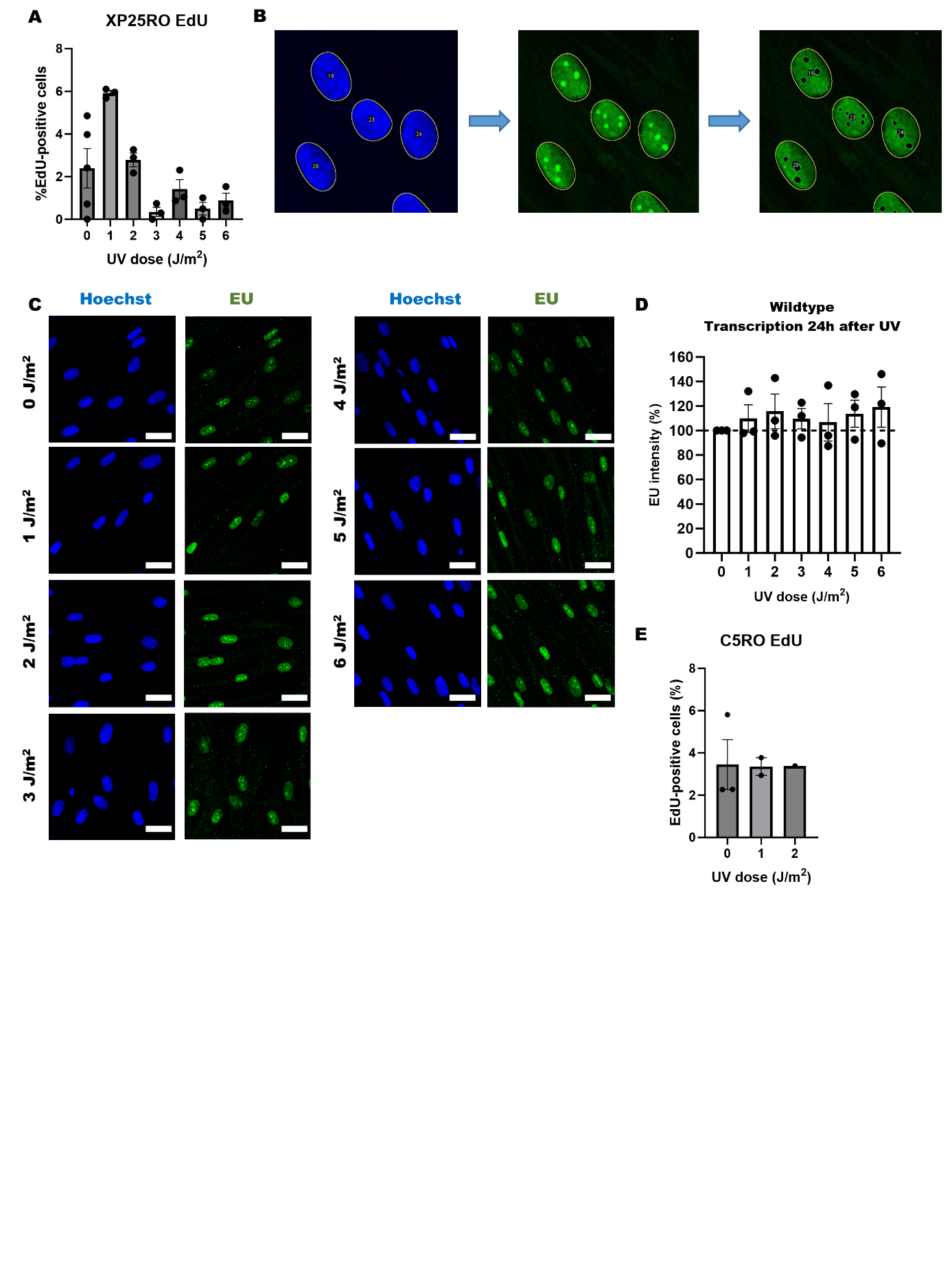


**Supplemental Figure 2: Transcription in wildtype fibroblasts and replication levels.** **(A)** Percentage of EdU-positive XP25RO cells 24 hours after UV (across three independent experiments). Error bars depict SEM of three experiments. **(B)** Visualization of the image analysis methods. In the first panel, nuclei are segmented based on the Hoechst channel and selected conform the criteria (size and circularity). Selected nuclei are circled in all three panels. Next, the selected nuclei ROIs are translated to the EU channel (second panel). Nucleoli are detected using threshold segmentation within the nuclei and subtracted from the EU channel (third panel). Finally, the mean EU intensity of the resulting nucleoplasm is measured. **(C)** Representative confocal images of C5RO (wildtype) cells treated with different UV doses (0-6 J/m^2^), stained with transcription marker EU (green) and DNA marker Hoechst (blue) 24 hours post-treatment. Scale bar = 30 μm. **(D)** Quantification of EU intensity in C5RO cells 24 hours after UV exposure (0-6 J/m^2^) across three independent experiments. Error bars represent SEM. **(E)** Percentage of EdU-positive cells per UV dose in C5RO (across three independent experiments). Error bars depict SEM of the three experiments.


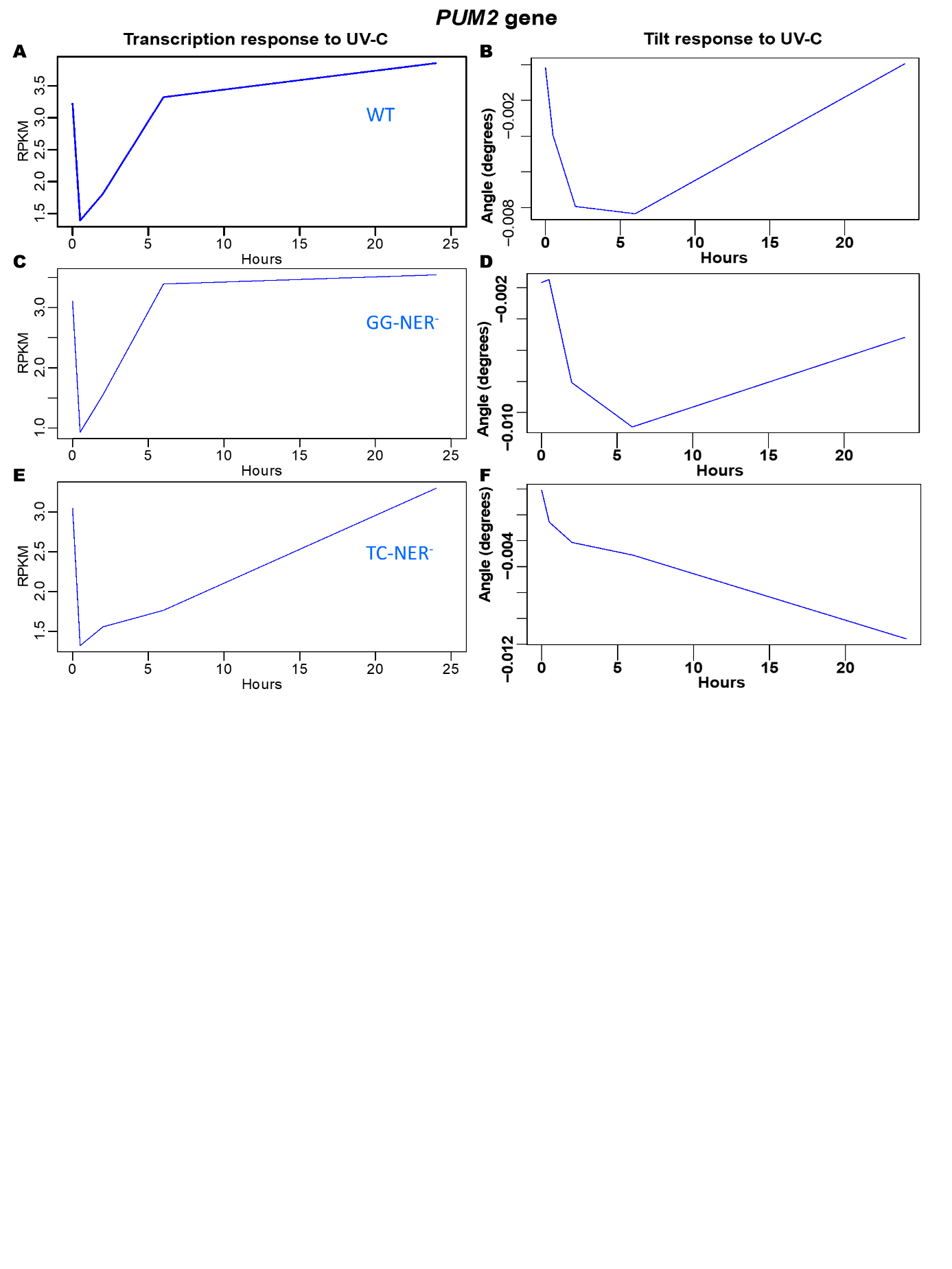


**Supplemental Figure 3: Transcription and tilt recovery for a representative long gene.** **(A)**–**(C)** Transcription levels of the *PUM2* gene (104 kb), measured in RPKM, at 0 (untreated control), 0.5, 2, 6, and 24 hours after 10 J/m² UV irradiation in **(A)** wildtype (HF1), **(B)** GG-NER-deficient (XPC-deficient; XP67TMA), and **(C)** TC-NER-deficient (CSB-deficient; CS1AN) human dermal fibroblasts (Andrade-Lima, Veloso, Paulsen, Menck, & Ljungman, 2015). **(D)**–**(F)** Transcription tilt (angle of the fitted line to RPKM read counts along the *PUM2* transcript) over the same time points in **(D)** wildtype, **(E)** GG-NER-deficient, and **(F)** TC-NER-deficient fibroblasts (Andrade-Lima et al., 2015).


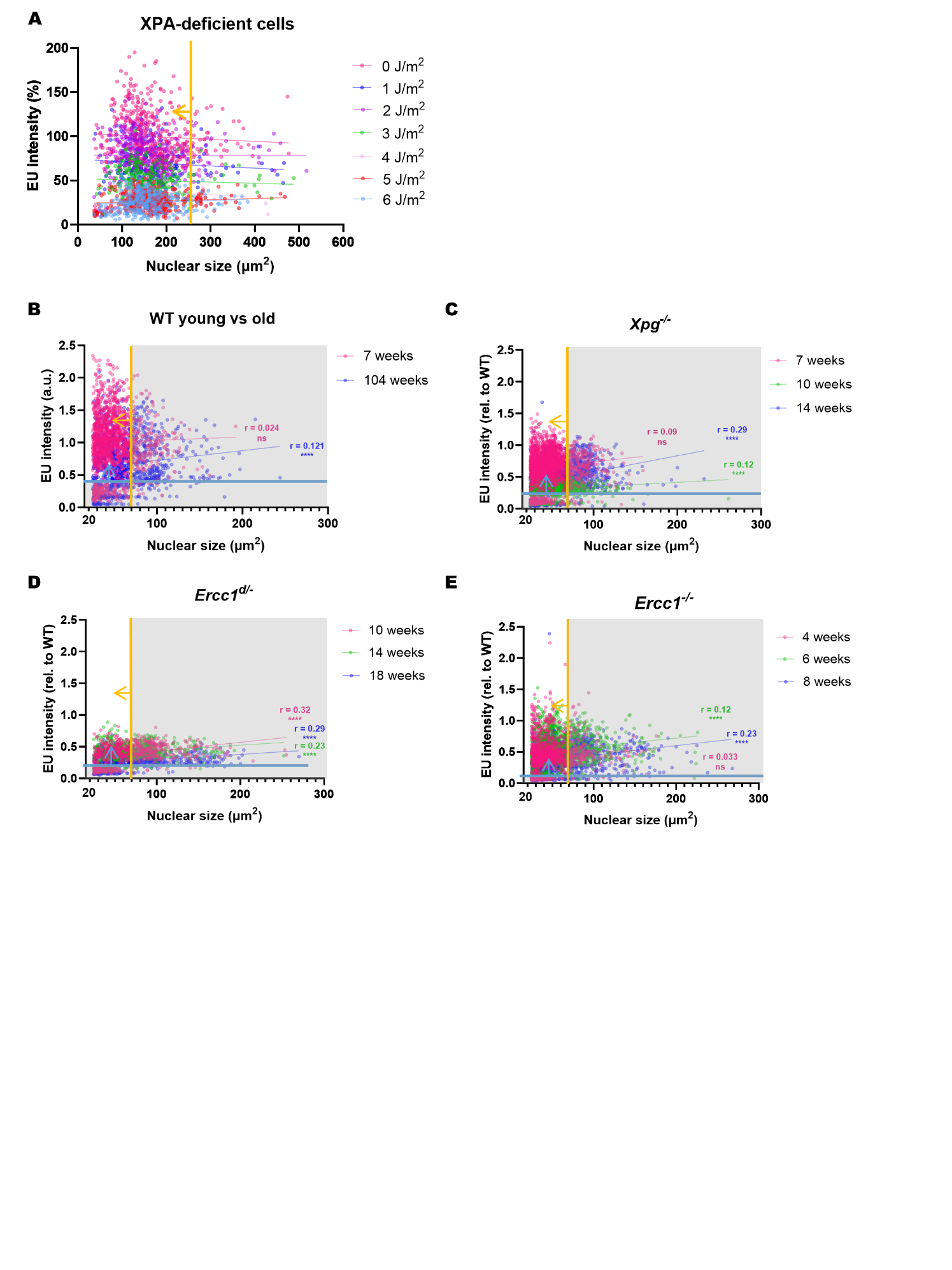


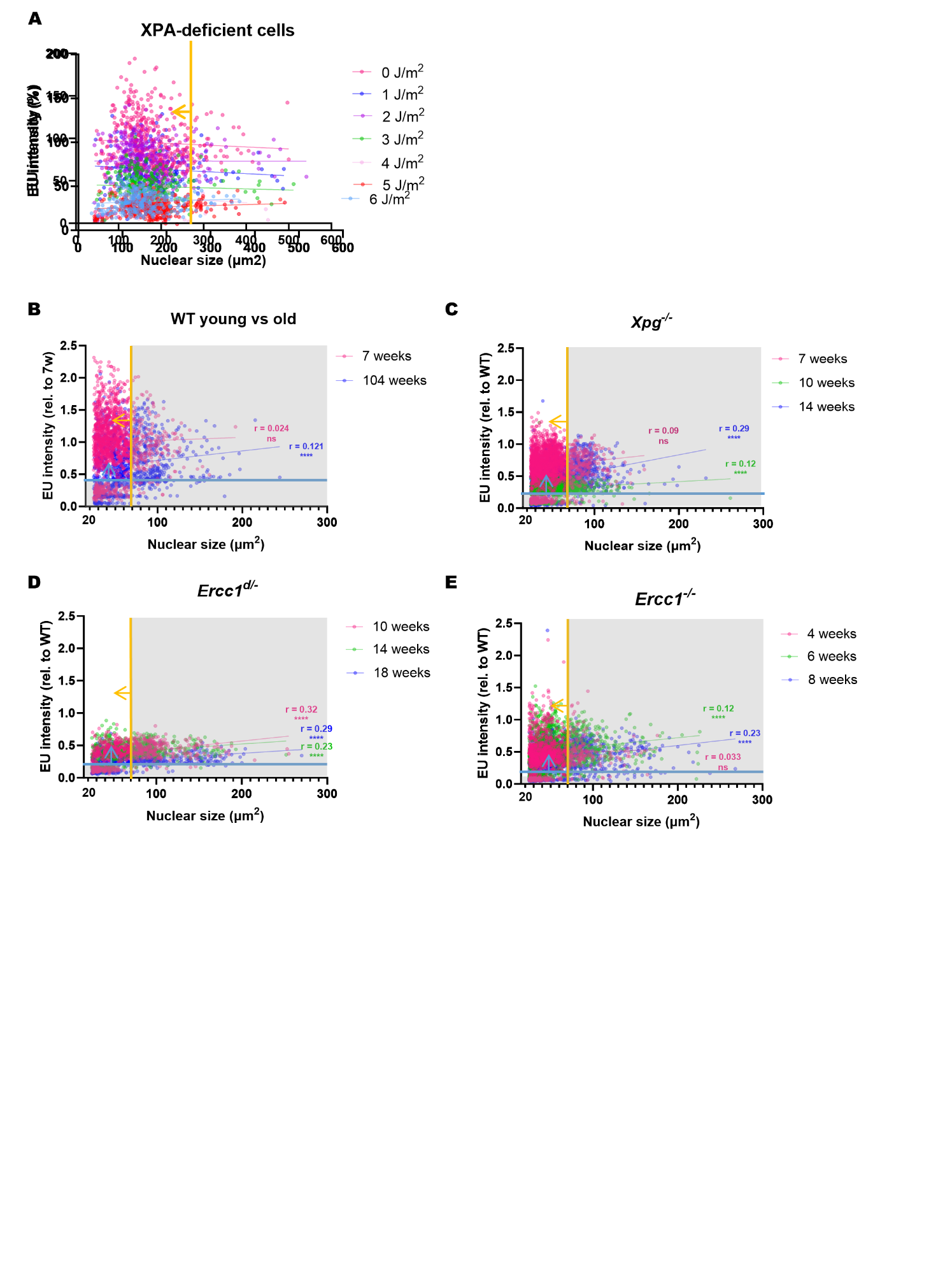


**Supplemental Figure 4: Correlation between nuclear size and EU signal. (A)** Scatterplot of nuclear size vs. mean EU intensity in the nucleoplasm in XPA-deficient human fibroblasts (XP25RO), normalized to WT of the same age. Each dot represents a cell; colored lines indicate per-sample correlations. Nuclei to the right of the orange vertical line were excluded as polyploid. **(B)–(E)** Same analysis in mouse liver cells from **(B)** wildtype, **(C)** *Xpg^-/-^*, **(D)** *Ercc1^d/-^*, and **(E)** *Ercc1^-^*^/-^ mice. Cells below the blue horizontal line (likely Kupffer cells) were excluded. R² values and statistical significance of the correlations are shown.


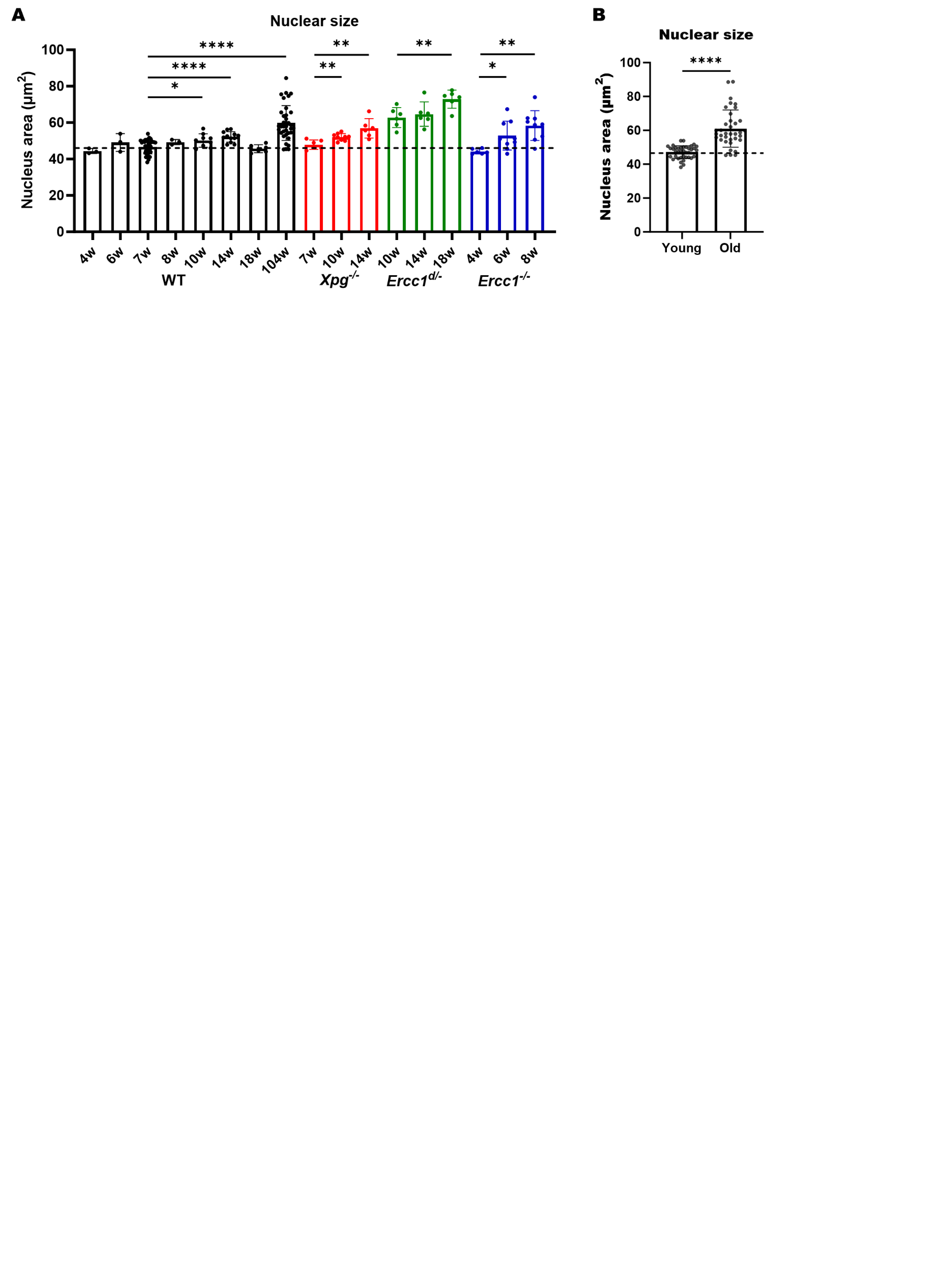


**Supplemental Figure 5: Nuclear size mouse hepatocytes.** **(A)** Average nuclear size in hepatocytes across all ages for wildtype, *Xpg^-/-^* , *Ercc1^d/-^* and *Ercc1^-^*^/-^ mice. Data points represent FOV averages; error bars indicate SD. **(B)** Average nuclear size in young (6–8 weeks) and old (104 weeks) wildtype liver sections, quantified via Hoechst staining, including data from our previous publication (19). Data points represent FOV averages; error bars indicate SD. Unpaired two-tailed Student’s *t*-test: * p≤0.05; ** p≤0.01; *** p≤0.001; **** p≤0.0001.

## Supplementary Tables

**Supplemental Table 1: Nascent RNA sequencing data human fibroblasts (C3RO, N=4).** For each gene, the table lists gene length in base pairs, normalized expression values across four biological replicates, and the average expression.

**Supplemental Table 2: Nascent RNA sequencing data young adult mouse liver (wt, 15 weeks, N=3).** For each gene, the table includes gene length in base pairs, normalized expression values across three biological replicates, and the average expression.
